# Physiotherapy Management of Bell's Palsy in an Elderly Patient: A Case Report

**DOI:** 10.7759/cureus.88045

**Published:** 2025-07-15

**Authors:** Uchechukwu B Abioke, Wonderful Anosike, Aliyu O Olaniyi, Ijeoma L Okwuowulu, Madhumati Mandal, Chisom Y Eze

**Affiliations:** 1 Physical Therapy, University of Benin Nigeria, Benin, NGA; 2 General Adult Psychiatry, Cumbria, Northumberland, Tyne and Wear NHS Foundation Trust, Newcastle Upon Tyne, GBR; 3 Geriatrics, Stepping Hill Hospital, Stockport NHS Foundation Trust, Stockport, GBR; 4 Clinical Sciences, College of Medicine University of Ibadan, Ibadan, NGA; 5 Medical Rehabilitation, Faculty of Health Sciences and Technology, University of Nigeria, Enugu, NGA

**Keywords:** bell’s palsy, case report, facial rehabilitation, kinesio taping, neuromuscular re-education

## Abstract

Bell’s palsy is a sudden-onset, unilateral facial paralysis most commonly regarded as idiopathic in origin. This case report discusses the physiotherapy management of a 75-year-old male with idiopathic Bell’s palsy. A structured physiotherapy regimen involving electrical stimulation, Kinesio taping, massage, and facial exercises was administered. Improvements were observed as the patient’s House-Brackmann score progressed from grade IV to III. Facial symmetry improved significantly, and muscle tone progressed from hypotonia to normotonia. Functional recovery, including the regained ability to drink water from a cup, along with notable improvements in oral motor functions such as chewing and articulation. Early intervention and a multidisciplinary approach combining medical and physiotherapy management facilitated recovery.

## Introduction

Bell’s palsy is a sudden-onset, unilateral facial paralysis caused by inflammation or compression of the facial nerve, also known as the seventh cranial nerve. This nerve is responsible for the motor control of facial expressions, and when inflamed, it results in weakness or complete loss of movement on one side of the face. Special attention is given to forehead muscle involvement to differentiate peripheral from central causes. This is because the forehead muscles receive input from both sides of the brain and so are preserved in central lesions [1]. It is primarily a diagnosis of exclusion, determined through clinical assessment.

Symptoms often appear suddenly, are progressive and can include facial drooping, inability to close the eye, and difficulty with speech or eating [1]. The exact cause of Bell’s palsy remains unknown in most cases, with up to 70% considered idiopathic, although several potential triggers have been identified. These include viral reactivations as well as underlying risk factors like diabetes, pregnancy, obesity, and hypertension [2]. The global incidence of Bell’s palsy is estimated to be around 20 to 30 cases per 100,000 individuals annually [3]. Medical management of Bell’s palsy involves anti-inflammatory strategies, with corticosteroids remaining the gold standard of treatment to reduce facial nerve inflammation, especially when initiated early. Addressing comorbidities like diabetes or hypertension is crucial, as these conditions may impair nerve healing [4].

Physiotherapy plays a vital role in improving functional outcomes, particularly in cases where full recovery is delayed. Techniques such as facial proprioceptive neuromuscular facilitation (PNF) and electrical stimulation have shown benefit in promoting symmetrical facial movement and reducing synkinesis [5]. The Mirror Effect Plus Protocol and structured facial exercises have demonstrated promising long-term outcomes. Additional supportive interventions like using goggles to protect the eye, facial massage, and educating patients on avoiding overuse or forced movements are also recommended. Overall, coordinated effort among the multidisciplinary team guarantees holistic and well-rounded patient care [6].

## Case presentation

The patient is a 75-year-old male who presented with a history of facial weakness. His symptoms began around June 2024 when he began to notice weakness of the left facial muscle, which resulted in drooling, slurred speech, and difficulty drinking from a cup. Over time, these symptoms led to visible deviation to the right due to left-sided weakness. His past medical history includes hypertension, diabetes mellitus, and peptic ulcer, which, over the years, were managed with antihypertensives, antidiabetics, and antacids.

The patient was placed on treatment that included corticosteroids to reduce facial nerve inflammation. Following medical stabilization, he was referred to the Physiotherapy Department in November 2024 for expert rehabilitation. On examination, he was alert, oriented, afebrile, and in no respiratory distress. His vital signs were stable (BP: 136/82 mmHg; PR: 101 bpm; blood glucose level: 6.3 mmol/L). Facial assessment revealed hypotonia on the left side and slight hypertonia on the right, with facial measurements of 12 cm and 11 cm, respectively. He had difficulty with oral functions such as drinking, chewing, and speaking. Outcome measures included a House-Brackmann (HB) grade of IV, a Facial Disability Index (FDI) score of 40%, and a Sunnybrook Facial Grading System score of 25%.

Clinical findings

Written informed consent was obtained from the patient. On inspection, facial asymmetry was observed with left-sided involvement. The nasolabial fold was absent on the affected side, the angle of the mouth was dropped, forehead wrinkles were not visible, and eye closure was incomplete. Bell’s phenomenon was present, and facial excursion was significantly reduced. Facial measurements were obtained using a flexible measuring tape, measuring from the tragus of the ear to the corner of the mouth on both sides. This comparative method helped assess the degree of facial asymmetry (Table 1).

**Table 1:** Timeline of events.

Events	Date
Onset of facial weakness	June 18, 2024
Date of presentation to the physiotherapy outpatient clinic	November 24, 2024
Date of follow-up	February 9, 2025

On initial examination, the patient’s House-Brackmann score was grade IV, indicating moderately severe facial nerve dysfunction. At the commencement of treatment, the Sunnybrook Facial Grading System yielded a score of 25, while the Facial Disability Index (FDI) was recorded at 40%, reflecting moderate functional impairment.

Differential diagnoses included cerebrovascular accident, Ramsay Hunt syndrome, Lyme disease, and Guillain-Barré syndrome. Ramsay Hunt syndrome was ruled out due to the absence of herpetic vesicles and ear blisters, and a lack of neurological symptoms supported the exclusion of stroke. Viral infection or reactivation was ruled out based on normal white blood cell count and differential, along with negative viral polymerase chain reaction (PCR) results.

Intervention

The patient was diagnosed with left-sided facial muscle impairment associated with Bell's palsy. He was placed on an eight-week rehabilitation protocol comprising progressively structured physiotherapy interventions, delivered thrice weekly in 45-minute sessions. The treatment plan aimed to enhance muscle function and improve facial symmetry. It included electrical muscle stimulation (EMS), facial massage, tactile stimulation, facial exercises, mirror therapy, and Kinesio taping. The patient was educated about his condition, and advice on lifestyle modifications was provided. He was advised to drink water with a straw to avoid spillage and encouraged to chew food on the affected side to strengthen the weakened masseter muscles. Ergonomic advice, such as wearing goggles for eye protection and taping the eyes at night to prevent dryness, was also given.

**Figure 1:**
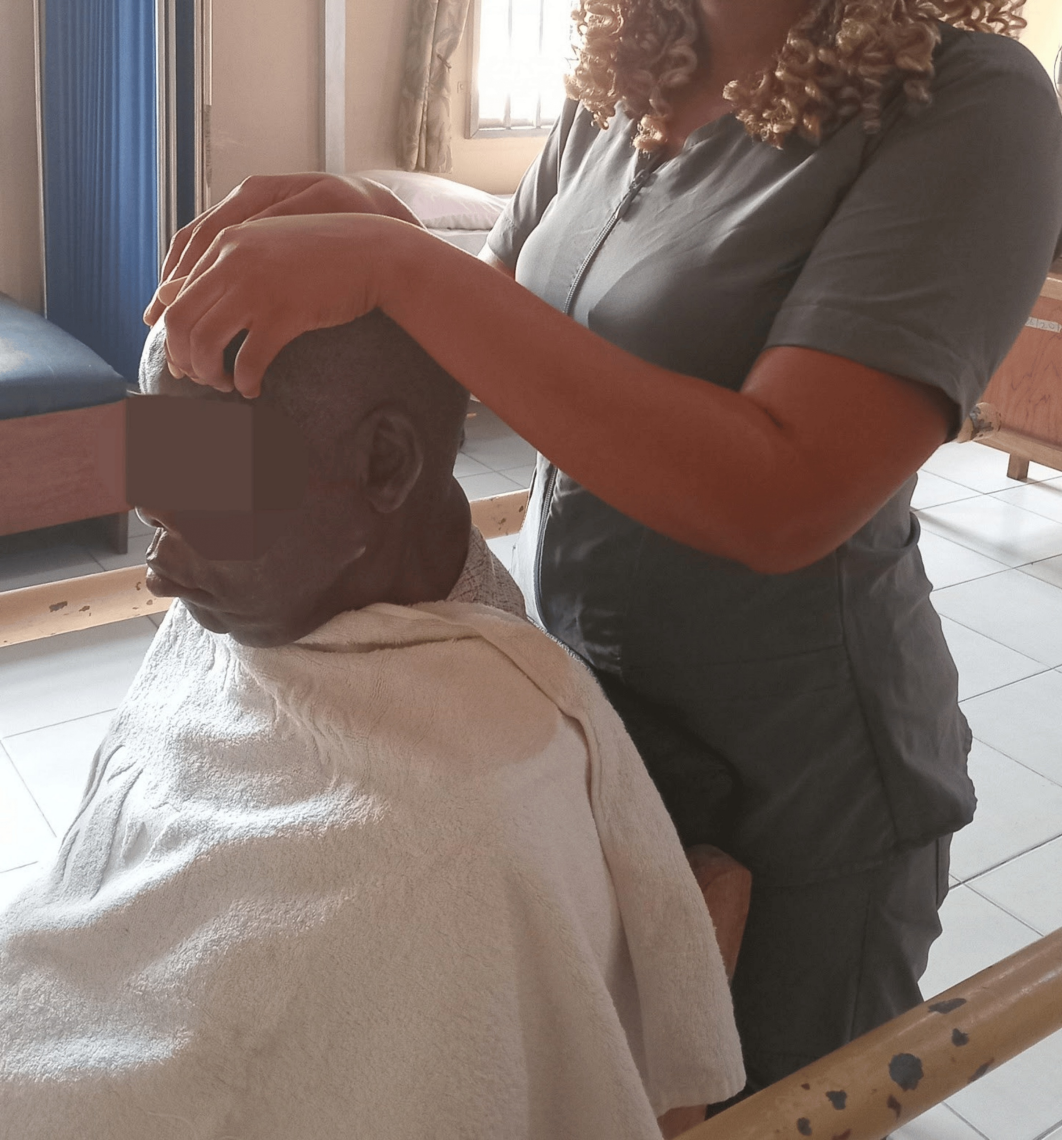
Application of the Kabat protocol beginning at the upper fulcrum (frontalis muscle). Resistance is applied to the unaffected (stronger) side to facilitate movement and muscle activation on the affected side, following diagonal and rotational PNF principles. PNF: proprioceptive neuromuscular facilitation.

The rehabilitation protocol incorporated multiple therapeutic techniques to enhance facial muscle function and recovery. Central to the approach was the Kabat technique, which was administered to three key facial regions: fronto-ocular, nasolabial, and perioral-mental areas. Each region received three sets of five to ten repetitions, conducted three times weekly as illustrated in Figure 1.

**Figure 2:**
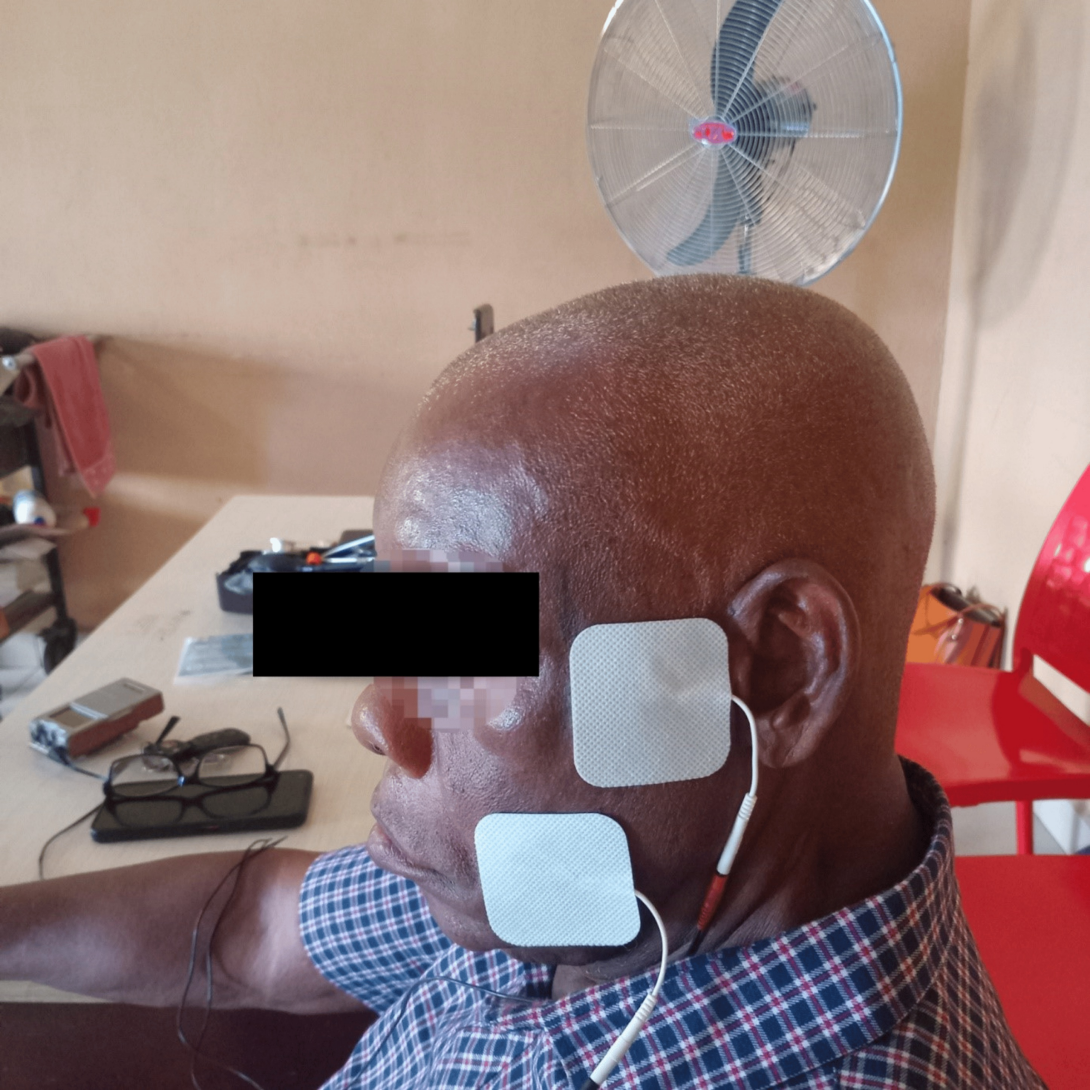
Application of electrical stimulation to the orbicularis oculi and orbicularis oris muscles to promote neuromuscular re-education and improve muscle tone. Parameters used: frequency (1-10 Hz), moderate intensity (2-5 mA), duration (15 mins), mode (NMES), pulse duration (150 µs), on: off time (five seconds on, 15 seconds off). NMES: neuromuscular electrical stimulation.

Facial exercises played a vital role in the regimen, which included exercises such as forehead wrinkling, smiling, eyebrow elevation, and lip puckering. They were performed in five repetitions, three times daily. Electrical muscle stimulation (EMS) complemented these exercises by targeting the orbicularis oris and orbicularis oculi muscles. Sessions lasted for 15-30 minutes, three times per week, utilizing low-frequency (1-10 Hz) and moderate-intensity (2-5 mA) settings, as shown in Figure 2.

**Figure 3:**
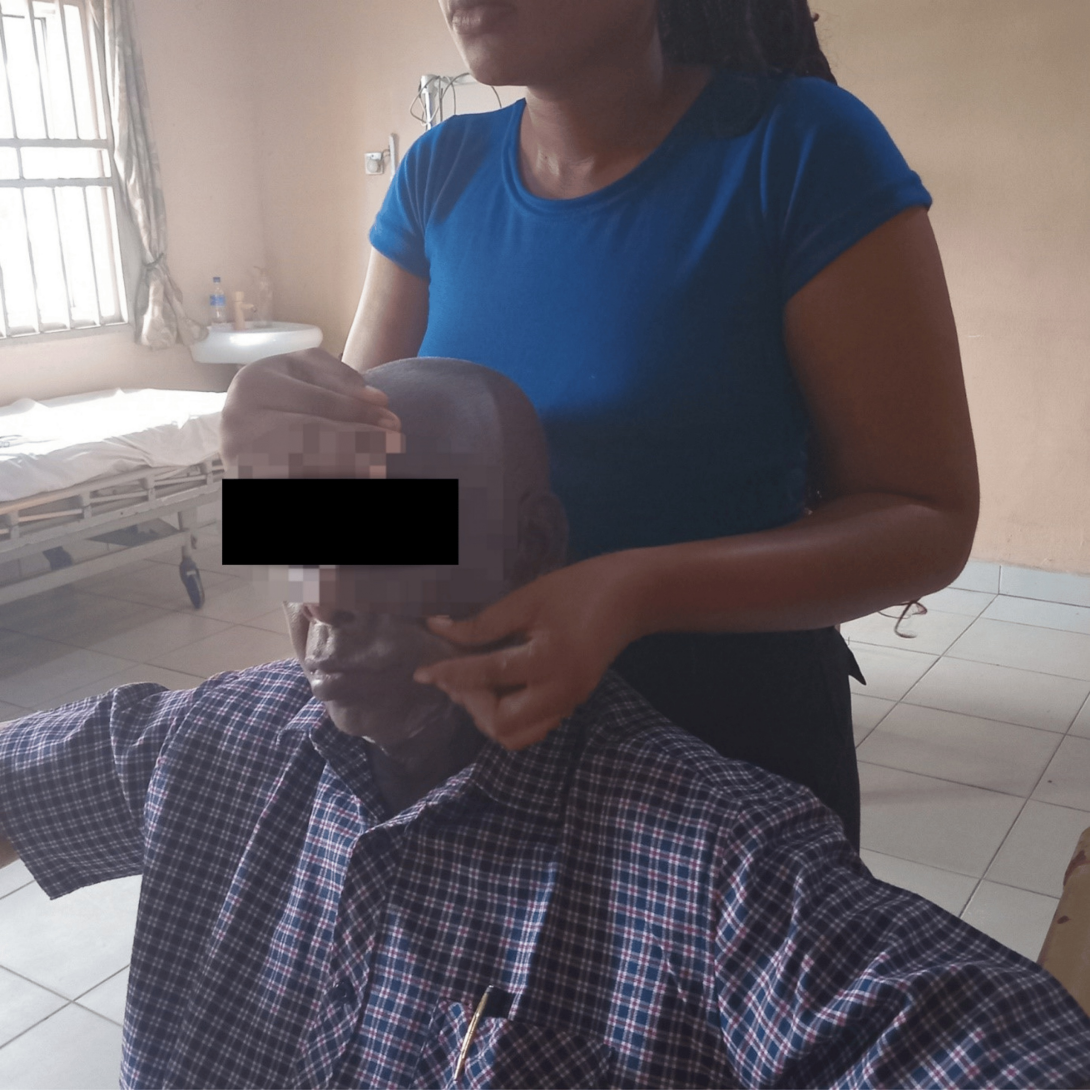
Tactile stimulation and facial massage (picking technique) are applied to facial muscles. Powder is used to reduce friction during manual stimulation, aimed at enhancing sensory feedback and improving circulation in affected areas.

Each therapy session also included facial massage and tactile stimulation, lasting approximately five to 10 minutes (Figure 3). Mirror therapy was incorporated to provide visual feedback and facilitate muscle retraining. Patients were encouraged to perform facial exercises, the Kabat technique, and Kinesio taping in front of a mirror to reinforce neuromuscular coordination.

**Figure 4:**
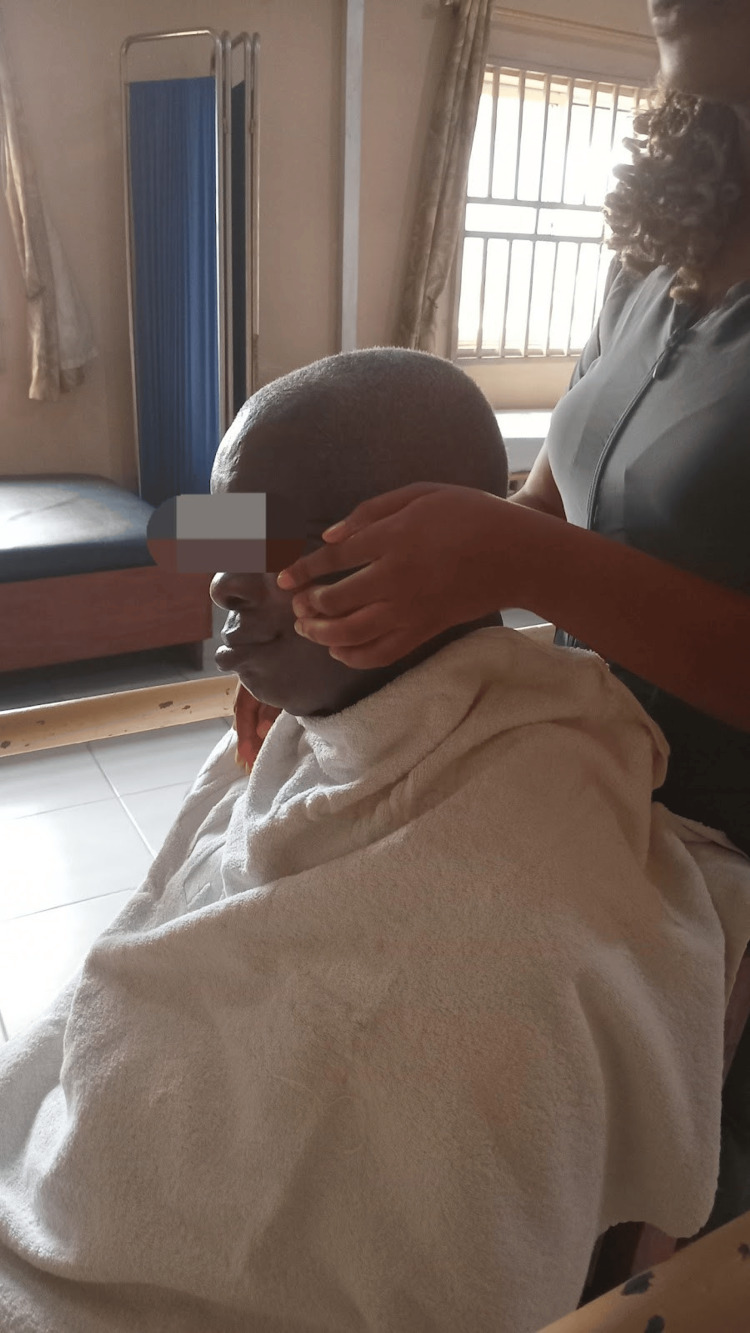
Proprioceptive neuromuscular facilitation (PNF) techniques are applied to facial muscles, incorporating diagonal and rotational movements to stimulate coordinated muscle activity and restore functional patterns.

Proprioceptive neuromuscular facilitation (PNF) to the facial muscles was also used as part of the rehabilitation process. PNF is used to promote the response of nerve impulses through the stimulation of proprioceptors in the body. The patient was also treated with this technique, as shown in Figure 4.

**Figure 5:**
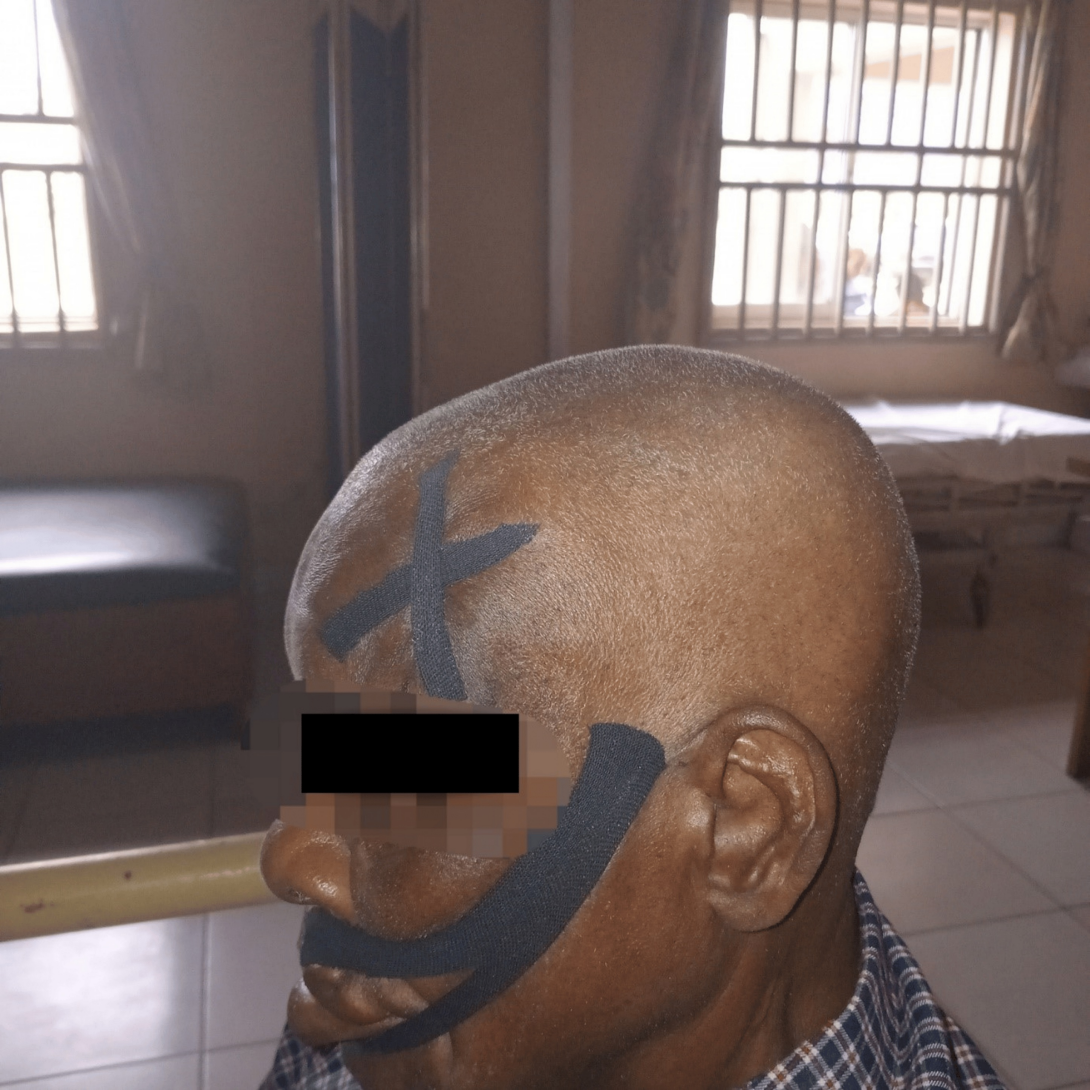
Kinesio taping applied to the frontalis, orbicularis oculi, and orbicularis oris muscles to facilitate muscle activation, provide proprioceptive input, and support functional alignment. Tape was applied following the natural orientation of each muscle: vertically from the eyebrows toward the hairline for the frontalis, circularly around the eye for the orbicularis oculi, and along the outline of the lips for the orbicularis oris.

Kinesio taping was applied strategically to support muscle strengthening, particularly on the affected side of the face. One strip was anchored around the orbicularis oculi and extended along its direction to provide support without restricting movement. Additionally, two smaller strips were applied to the forehead to engage the frontalis muscle. For modified eyebrow lifting exercises, a small section of the forehead tape was intentionally left unattached, allowing for active participation in the rehabilitation program, as illustrated in Figure 5.

**Figure 6:**
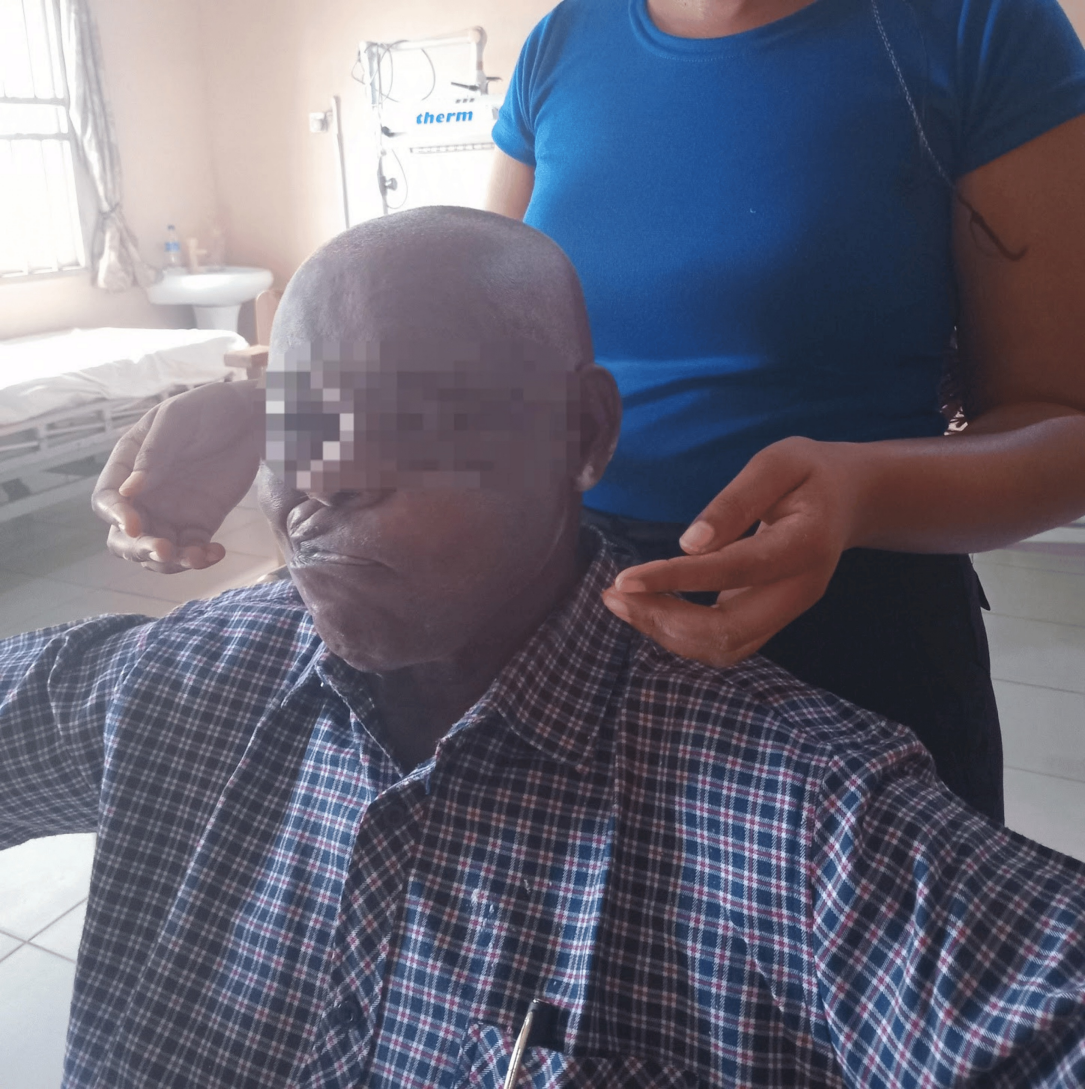
Facial exercise involving blowing exercise (as if to fill a balloon) to strengthen the orbicularis oris, buccinator, and other perioral muscles. This exercise promotes improved muscle tone, oral competence, and coordination for functional facial expressions.

To reinforce clinical interventions, a structured home program was introduced. This included facial exercises, such as blowing a balloon, sucking in the cheeks, smiling, frowning, and lip puckering, as shown in Figure 6. These activities were performed consistently to strengthen motor control and facilitate muscle activation. Across the eight weeks, frequency remained stable while intensity and repetitions were progressively increased to facilitate motor recovery and functional improvement.

Outcome and follow-up

**Figure 7:**
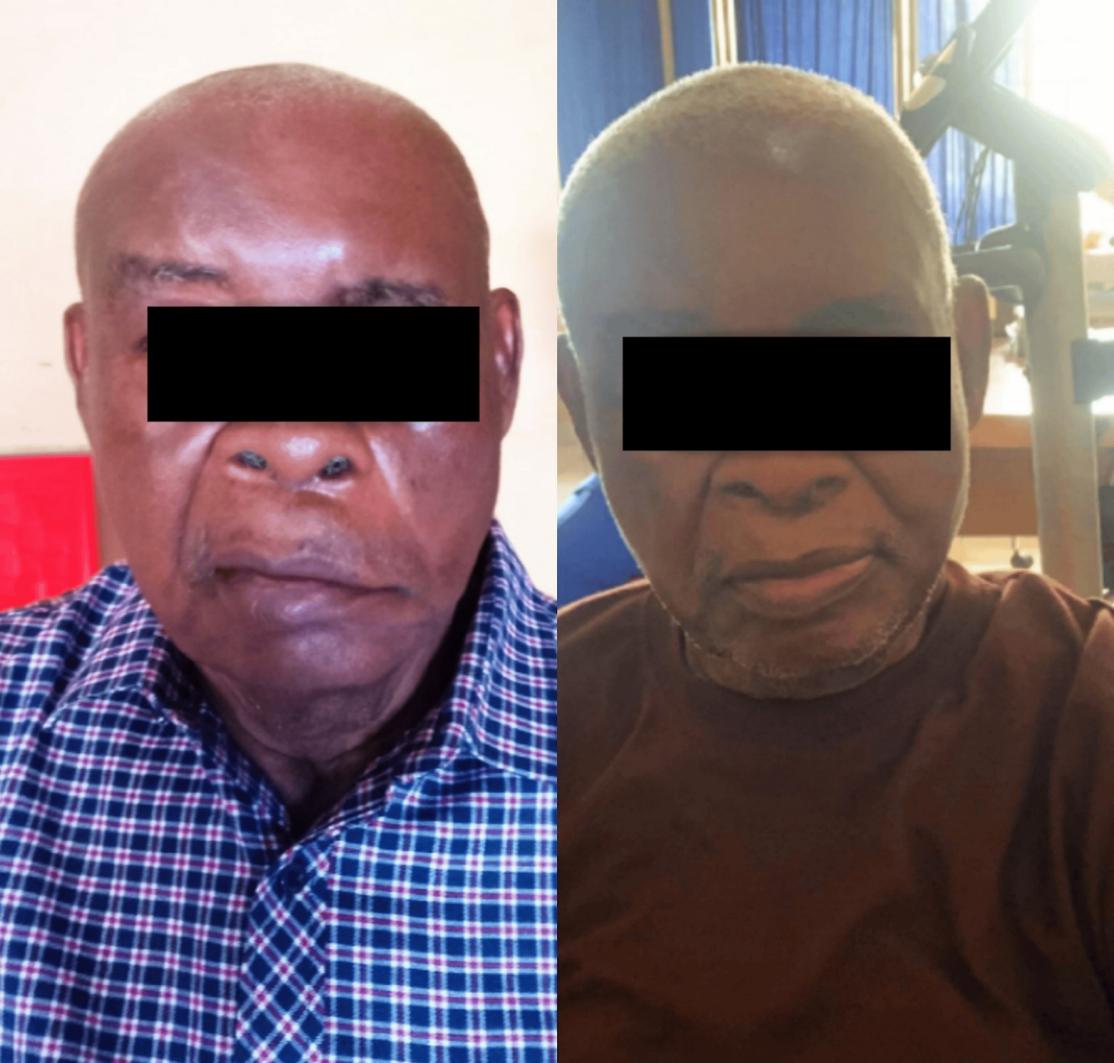
LT: Pre-intervention photograph. RT: Post-intervention photograph showing marked improvement in left facial muscle function. Notable changes include a more defined nasolabial fold, reduced eyebrow droop, improved facial symmetry, the ability to close the eyelid, and reduced synkinesis. Facial excursion also improved, indicating greater range and coordination of facial movements. LT: left; RT: right.

Following physiotherapy sessions, the patient showed notable improvements. Follow-up was done weekly. Table 2 displays the results of the outcome measures taken before and after treatment. The House-Brackmann score improved from grade IV to grade III, indicating better facial muscle function. The Facial Disability Index (FDI) increased from 40% to 70%, reflecting an improvement in his ability to perform daily facial tasks. Additionally, the Sunnybrook Facial Grading System score improved from 25% to 60%, suggesting better symmetry and voluntary muscle movement on the affected side. Tape measurement for facial asymmetry decreased from an initial 12.5 cm to 12.0 cm, showing improved muscle tone and facial alignment. These improvements allowed the patient to resume basic oral functions like drinking from a cup, chewing, and speaking more clearly. Overall, there was a significant improvement in the patient after the rehabilitation, as shown in Figure 7. Also, the recovery was facilitated by the collaboration between ENT specialists, physiotherapists, and other healthcare providers who were involved in the management of this patient. To support clinical documentation and facilitate progress monitoring, pre-treatment photographs were taken, as illustrated in Figure 7.

**Table 2:** Outcome measures pre- and post-rehabilitation.

Outcome measure	Pre-rehabilitation	Post-rehabilitation
House Brackmann Scale	IV	III
Facial Disability Index	40%	70%
Tape rule	Rt: 12.0 cm; Lt: 12.5 cm	Rt: 12.0 cm; Lt: 12.0 cm
Sunnybrook Score	25%	60%

## Discussion

This report reiterates the importance of early, multidisciplinary care in managing Bell’s palsy. Physiotherapy played a key role in facilitating recovery by improving muscle strength and coordination. This aligns with the randomized controlled trial by Ferreira et al. in 2015, which found that combining physiotherapy with medication led to better outcomes than drug therapy alone [6].

Furthermore, a study by Martineau et al. (2022) on the Mirror Effect Plus Protocol demonstrated significant long-term improvements in facial function, sustained up to one year post-treatment [7]. In the patient presented in this case report, notable progress was observed in weeks, particularly in the frontalis and perioral regions. Although the exact protocol was not employed, our findings suggest that a personalized, early-intervention approach may offer comparable benefits in a real-world clinical setting. The incorporation of electrical stimulation and Kinesio taping alongside traditional facial exercises appeared to enhance outcomes, a finding that echoes the work of Marotta et al. (2020), who reported favorable results with electrical stimulation in patients with unresolved Bell’s palsy [8]. 

In line with the updated systematic review by Khan et al. (2022), which supports the efficacy of facial exercises in the management of Bell’s palsy, the patient demonstrated notable improvement with a structured and progressive exercise regimen [9]. The intervention also incorporated individualized strategies, such as modified eyebrow lifting using Kinesio tape, which appeared to enhance both patient engagement and adherence. Surgical interventions, like those outlined by Gantz et al. (1999), were not necessary in this case, as the patient responded favorably to conservative treatment. This underscores the importance of early, personalized, and comprehensive care in optimizing functional recovery while minimizing the need for invasive procedures [10].

The impact of comorbidities on recovery was also carefully considered. The patient had diabetes, a condition known to potentially delay nerve regeneration and healing. Warner et al. (2022) emphasize that diabetes and other systemic illnesses can affect the prognosis of Bell's palsy by impairing microvascular circulation and reducing the body's ability to repair nerve damage. This understanding guided a more cautious treatment plan. The timing and dosage of corticosteroids and blood glucose levels were closely monitored throughout treatment, and follow-up was greatly ensured [11]. The treatment approach was tailored and dynamic. The intensity of electrical muscle stimulation (EMS) was initially kept low and progressively increased based on tolerance. Exercise frequency was modified to allow rest days, and an additional clinic visit was added weekly to ensure close monitoring and timely intervention. Modalities, such as EMS, facial massage, tactile stimulation, facial exercises, mirror therapy, and Kinesio taping, were combined to promote recovery.

Quantitatively, the patient demonstrated marked improvement across all outcome measures. The Sunnybrook Facial Grading System (SB-FGS) score increased from 25% to 60%, exceeding the minimal clinically important difference (MCID) of approximately 13 points, which is considered the threshold for meaningful clinical improvement in facial function [12]. The House-Brackmann (HB) score improved from grade IV to grade III, indicating enhanced gross motor function. Similarly, the Facial Disability Index (FDI) rose from 40 to 70, reflecting substantial gains in both physical and social aspects of facial disability. Together, these outcomes highlight not only measurable functional recovery but also the patient's improved quality of life.

The correlation across these outcome measures strengthens the clinical interpretation: the upward trend in SB-FGS paralleled the patient's improved FDI score and reduced HB grade, demonstrating both objective and subjective recovery. Visual documentation (Figure 7) reinforced these findings, showing enhanced facial symmetry, more pronounced nasolabial folds, improved eyelid closure, and reduced synkinesis. The patient also reported feeling more confident and expressive during social interactions, echoing the clinical and functional improvements. In summary, this case emphasizes the clinical value of adapting treatment to each patient’s specific needs. The integration of validated outcome measures, MCID-based interpretation, and patient-reported insights supports the effectiveness of a well-rounded, evidence-based rehabilitation approach for Bell's palsy.

Limitations

One notable limitation of this case report was the variability in the quality and adhesive strength of the Kinesio tape used during the intervention. Initially, a brand with suboptimal adhesive properties was applied, which led to premature detachment of the tape from the patient’s face before the next treatment session. This may have affected the consistency and potential effectiveness of the taping technique in the early phase of treatment. Subsequent sessions incorporated a higher-quality tape, but this inconsistency is acknowledged as a limitation in the overall management approach.

## Conclusions

Bell's palsy presents both diagnostic and therapeutic challenges, and it is essential to thoroughly evaluate and rule out other potential diagnoses to ensure accurate treatment planning and optimal patient outcomes. This case report demonstrates the significant improvements that can be achieved when patients receive early, coordinated, and multidisciplinary care. Timely physiotherapy, provided alongside medical therapy, played a critical role in the patient's progress.

The patient was placed on a structured conservative physiotherapy plan that included facial exercises, electrical muscle stimulation, Kinesio taping, mirror therapy, and a guided home program. Over time, he regained facial symmetry, muscle strength, and voluntary control, eventually performing targeted facial movements without assistance. Beyond the physical gains, the patient also reported restored confidence and emotional well-being. Although results from a single case cannot be broadly generalized, this report contributes to the growing body of evidence supporting individualized, conservative rehabilitation for Bell's palsy. It reinforces the importance of early diagnosis, prompt initiation of physiotherapy, and collaborative medical care.

## References

[1] Eviston TJ, Croxson GR, Kennedy PGE, Hadlock T, Krishnan AV (2015). Bell's palsy: aetiology, clinical features and multidisciplinary care. J Neurol Neurosurg Psychiatry.

[2] Singh A, Deshmukh P (2022). Bell's palsy: a review. Cureus.

[3] Mustafa AHK, Sulaiman AM (2018). The epidemiology and management of Bell's palsy in the Sudan. Open Dent J.

[4] De Seta D, Mancini P, Minni A (2014). Bell's palsy: symptoms preceding and accompanying the facial paresis. Sci World J.

[5] Somasundara D, Sullivan F (2017). Management of Bell's palsy. Aust Prescr.

[6] Ferreira M, Marques EE, Duarte JA, Santos PC (2015). Physical therapy with drug treatment in Bell palsy: a focused review. Am J Phys Med Rehabil.

[7] Martineau S, Rahal A, Piette E, Moubayed S, Marcotte K (2022). The "Mirror Effect Plus Protocol" for acute Bell's palsy: a randomized controlled trial with 1-year follow-up. Clin Rehabil.

[8] Marotta N, Demeco A, Inzitari MT, Caruso MG, Ammendolia A (2020). Neuromuscular electrical stimulation and shortwave diathermy in unrecovered Bell palsy: a randomized controlled study. Medicine (Baltimore).

[9] Khan AJ, Szczepura A, Palmer S (2022). Physical therapy for facial nerve paralysis (Bell's palsy): an updated and extended systematic review of the evidence for facial exercise therapy. Clin Rehabil.

[10] Gantz BJ, Rubinstein JT, Gidley P, Woodson GE (1999). Surgical management of Bell's palsy. Otolaryngol Head Neck Surg.

[11] Warner MJ, Hutchison J, Varacallo M, Busby TH (2022). Bell palsy (nursing). StatPearls (Internet).

[12] Ross BG, Fradet G, Nedzelski JM (1996). Development of a sensitive clinical facial grading system. Otolaryngol Head Neck Surg.

